# Sociocultural context of exclusive breastfeeding in Africa: A narrative review

**DOI:** 10.1002/hsr2.2115

**Published:** 2024-05-12

**Authors:** Jimoh Amzat, Kafayat Aminu, Brisca Matankari, Abbas Ismail, Bello Almu, Kehinde Kazeem Kanmodi

**Affiliations:** ^1^ Department of Sociology Usmanu Danfodiyo University Sokoto Nigeria; ^2^ Department of Sociology University of Johannesburg Johannesburg South Africa; ^3^ Centre for Child and Adolescent Mental Health University College Hospital Ibadan Nigeria; ^4^ Department of Sociology Umaru Musa Yar'adua University Katsina Nigeria; ^5^ School of Health and Life Sciences Teesside University Middlesbrough UK; ^6^ Faculty of Dentistry University of Puthisastra Phnom Penh Cambodia; ^7^ School of Dentistry University of Rwanda Kigali Rwanda; ^8^ Cephas Health Research Initiative Inc Ibadan Nigeria

**Keywords:** Africa, exclusive breastfeeding, global public health, mothers, review, sociocultural

## Abstract

**Background and Aims:**

Although exclusive breastfeeding (EBF) has many benefits, it is not commonly practiced in several countries as a result of context‐specific challenges. EBF practice is thus a global health discourse because over 200 million children suffer from malnutrition despite the abundance of human milk. The risk of starvation remains very high among African children with less than 40% of them exclusively breastfed. In Africa, the adoption or nonadherence to EBF is a sociocultural issue. Hence, this narrative review examined the sociocultural context of EBF in the region.

**Methods:**

PubMed, Google Scholar, and Scopus were searched using keywords related to EBF and Africa. Relevant data from selected studies were synthesized narratively and reported using a structured narrative format.

**Results:**

EBF is strongly rooted in every culture and is a culturally determined behavior. Some believe that colostrum is dirty and harmful to newborns and, thus, needs to be purified. Despite the belief that human milk is the best food for babies, mothers often complement human milk with other foods right from birth because of perceived lactation inadequacy. Most African mothers believe in prelacteal feeding to help cleanse the infant's gastrointestinal tract for digestion, quench thirst, flush the bladder and help the mother to rest after childbirth before breastfeeding (BF) is initiated. The role of significant others was equally found important in the decision and duration of BF. The availability of family support (especially from grandmothers and husbands) reportedly encouraged EBF in Africa. The duration and exclusivity of BF in Africa are negatively associated with demographic variables like young age, low level of education, being unmarried, low income, out of employment, and parity (first‐time mother).

**Conclusion:**

While there have been some efforts and policies to improve EBF, it is important to consider context‐specific challenges and sociocultural factors. There is a need for more deliberate efforts to encourage mothers through the implementation of effective best practices concerning EBF in Africa.

## 1 INTRODUCTION

Breastfeeding (BF), a source of human milk, has been recommended for newborns globally as the best source of nutrition for healthy growth and development.^1^ Exclusive breastfeeding (EBF) implies giving the newborn only human milk without any complementary solids or liquids except oral rehydration solution, drops or sirups consisting of vitamins, minerals, supplements or medicine.^2^ The justification for the World Health Organization's (WHO's) recommendation of EBF for up to 6 months is that it is better than any other product given to a child.^3^ After 6 months, mothers are advised to introduce other foods along with human milk for up to 2 years.^2^, ^4^ Although EBF has many benefits, it is not commonly practised in several African countries as a result of context‐specific challenges.^3^, ^5^, ^6^, ^7^ The practice of EBF in many low‐ and middle‐income^8^ and global settings^9^ is low. Thus, EBF is a global health discourse because over 200 million African children suffer from malnutrition which is the second leading cause of death after malaria^10^ despite the abundance of human milk. The risk of starvation for children is still very high in Africa.

In regions with high levels of food insecurity and malnutrition, mothers may struggle to meet their own nutritional needs, which can impact their ability to breastfeed effectively and provide adequate nutrition to their infants. For the newborn, it is expected that the availability of human milk will moderate their malnutrition but it is not as much as it should be due to nonadherence to EBF. The prevalence of EBF is generally low in Africa.^5^, ^11^ Approximately 53% of newborns received BF within the first hour of birth between 2015 and 2021, which falls short of the intended goal of 70%.^9^ Interestingly, 67% of women sustain BF for a minimum of 1 year within the period. Bhattacharjee et al.^11^ noted that only 37% of African children were exclusively breastfed in 2017. United Nations Children's Emergency Fund^12^ estimated an improvement in 2021 documenting that 44% of African children were EBF in 2021, with variations across the regions of Africa: North Africa (32%), Southern Africa (33%), West Africa (35%), East Africa (59%), and Central Africa (44%). While the rates are unacceptably low, they represent improvement due to some concerted efforts from different stakeholders. There are also internal variations across the countries with EBF less than 20% in some countries including Côte d'Ivoire^13^ and Nigeria. It is important to note that EBF is a global challenge as it is relatively low in Western societies too.^14^

The main problems are about early introduction of unhealthy foods and unhealthy feeding practices which hinder EBF. The adoption or nonadherence to EBF is a sociocultural issue in Africa. Hence, Oyelana et al.^5^ observed that the adoption of EBF requires more sociocultural processes rather than technical processes. Extended family members and grandmothers have significant roles in childcare, and they have a powerful influence on innovative decision‐making about EBF.^5^ Cultural beliefs and traditions in some African communities may discourage EBF. For example, some cultures promote the early introduction of complementary foods or water, which can interfere with EBF. Cultural factors play a significant role in influencing EBF practices. These cultural factors can vary widely from one community to another and can either support or hinder EBF. Therefore, this paper examines local understanding and sociocultural beliefs relating to EBF for children in Africa.

## 2 METHODS

This narrative review provides an overview of the sociocultural context of EBF practices in the African region. The steps followed in conducting the review are highlighted as prescribed by Demiris and colleagues.^15^

We conducted a comprehensive search of electronic databases such as PubMed, Google Scholar, and Scopus using keywords related to EBF (“exclusive breastfeeding,” “breastfeeding practices,” “infant feeding”) and geographical location (“Africa,” “sub‐Saharan Africa”). The reviewers identified additional relevant sources through manual searches of reference lists from selected articles. Studies eligible for inclusion in the review are those focused on EBF practices in Africa. We also selected publications irrespective of the study design. That is, studies that reported using qualitative, observational, surveys, and intervention research designs were included in the review. Articles published in English language from 1991 to 2023 were included. However, studies on nonhuman subjects or conducted outside the African continent were excluded.

After reviewing the abstracts and full texts of selected publications, relevant data, including sample characteristics, geographical location, BF initiation and duration rates, and factors influencing EBF practices in Africa were extracted from eligible studies. Findings were summarized and narratively synthesized and incorporated into the text. Themes related to EBF practices in Africa, such as understanding and beliefs about BF, sociocultural factors, or issues responsible for poor adherence to EBF, beliefs regarding the need for complementary feeding, policy or program interventions and best practices that work to promote EBF were identified. Quantitative data, such as prevalence rates were descriptively reported. The results are presented in a narrative format, supported by descriptive statistics where applicable.

## 3 RESULTS

### 3.1 Local understanding and cultural beliefs about BF for children

The cultural norms, beliefs and traditions related to BF exist in every culture. This explains why EBF is strongly rooted in culture and is a culturally determined behavior.^16^, ^17^ Different beliefs related to BF were identified and grouped into subthemes. While some of these beliefs have done a lot of harm others have been beneficial to child health. BF beliefs in different social groups have been documented to reveal some general and unique philosophies associated with EBF and infant feeding. There is a common notion that BF is easy and more like an instinct while it was found to require support, practice and time for mothers to master the correct position for babies to latch and feed properly.^18^ The practice of bathing the mother and child before the initiation of BF is a common practice in Africa.^19^

A report by Oyelana et al. indicates that the belief that BF is a standard practice for infant feeding has been in existence for so long in traditional African culture.^5^ According to them, before the introduction of EBF, mothers living in Africa perceived BF as a means of establishing a profound bond between mother and child. Additionally, there exists a prevalent belief among Africans in the potency of breastmilk (BM), with some attributing the ability to bestow blessings or curses upon a child to its power, hence expectant mothers often anticipate BF with a sense of pride.^5^ According to Agunbiade and Ogunleye,^20^ some grandmothers viewed BF as an investment in a child's life while Davies‐Adetugbo in a different study reported that mothers understood BF would help them in child spacing.^21^

However, different studies found that nursing mothers held many wrong beliefs about BF.^22^, ^23^ Mothers in Ethiopia feared that evil eyes affect BM supply when a child is nursed in public places, while some feared that BF mothers could develop pain if the child burped while nursing. Additionally, pregnancy is believed to make BM impure, and BM has a bad odor. There is also a widespread belief that the consumption of sugary foods/drinks by mothers may cause a breastfed child to develop stomach pain, known as *chango/mekakuu*.^22^ Hence, investigations in Africa provide evidence that awareness and knowledge about the importance of BF did not produce positive attitudes towards EBF but rather cultural and religious beliefs had more influence on infant feeding practices.^5^

#### 3.1.1 Cultural beliefs about colostrum and prelacteal feeding

In Africa, most cultures are said to identify colostrum to be not beneficial to newborns due to the widespread limited knowledge about its health benefits. As Asaro^24^ noted, colostrum is regarded as “bad milk” and harmful to the infant in some African cultures and mothers tend to dispose of it. Such practices have been reported among mothers in Ethiopia where the tradition of colostrum avoidance is practised by mothers in the North‐Eastern part of the country because they perceived it as dirty and not good due to its color and consistency. They thought that colostrum caused diseases and abdominal cramps in babies hence they habitually discard it.^25^ Similarly, a previous study in a rural community indicated that women perceived colostrum as dirty and harmful and mothers likened it to “pus.” They also regarded expressed human milk as easy to get poisoned, infected and could be bewitched by the evil ones.^21^

Due to the belief that colostrum is dirty and a potential source of diseases for infants, some family traditions/cultures require that new mothers take herbs to make breast milk edible for infants. This practice was documented among the Fulani where the breast is rubbed with a concoction of herbs and cheese to purify the BM before BF is initiated. It is noteworthy that the colostrum is fed to the baby in subsequent births after the breast is washed with herbs.^19^

Prelacteal feeding, which refers to any fluid provided to an infant before the commencement of BF,^26^ is believed to fortify or protect the child.^19^ Prelacteal food is introduced in Ethiopia to prevent “evil eye” and illness and to “clean infant's stomach.”^27^ Some mothers likewise considered meconium harmful and introduced prelacteal feed like saline and herbs to “cleanse” the infant and remove the meconium. After a few weeks of life, babies are introduced to herbs like *ekyogero* and *ensugo* to give the baby satisfaction and fortify them against certain childhood and pregnancy‐related diseases they have been exposed to before birth.^28^

Depending on the belief or worldview, it is similarly common to use prelacteal food like holy water, Arabic writing, honey, and dates. Islamic faith was found to have influenced this practice as the nursing mothers revealed that their significant others were advised to feed the infant with them by their faith leaders.^19^ Among mothers in Kenya, colostrum, characterized by its thick and yellowish or watery consistency, is often viewed unfavorably due to its appearance, differing from that of regular milk. Also, within the Sukuma community, the practice of feeding newborns colostrum is discouraged and considered taboo.^23^ This according to the investigators supports the general belief in the community that mother's milk is impure and could be harmful for up to 3 days.^23^, ^29^ Mothers introduced alternative feed like animal milk, boiled water, honey, and washouts from Arabic inscriptions on slates to their children during the time they wait and expect the breast milk to become clean and edible for the baby.^29^ In Uganda, mushroom soups and herbs are fed to infants for the first 2 weeks of life as a form of protection against abdominal cramps, and other ill health.^28^

### 3.2 Beliefs and practices affecting EBF

EBF for 6 months is often perceived as hindering the introduction of complementary foods. In specific ethnic communities, notably the Luo and Luhya, there exists a belief that if a mother engages in extramarital relationships with men other than the baby's father, her breast milk becomes tainted. This behavior is seen as a bad omen or curse referred to as *chira*, and continuing to breastfeed under such circumstances is believed to carry the risk of harming the baby, possibly leading to their death.^23^ Another finding reported is the notion that new pregnancy contaminates breast milk thus rendering it harmful to the infant, this makes lactating mothers who become pregnant unexpectedly stop BF.^29^

Furthermore, the belief that EBF is unsafe for the infant was reported among Yoruba and Bini mothers.^30^ Due to this, they feed water to the infant to quench thirst, stop hiccoughs, and promote normal growth. Some African mothers also feed their infants with herbal drinks and ritual fluids due to the perceived medicinal benefits to the infants.^21^, ^30^ Despite the belief that breast milk is the best food for babies, mothers reportedly complemented BF with water and tea right from birth because they viewed lactation as often inadequate while EBF is perceived as dangerous to the child.^21^ It was revealed that some mothers have faith in such prelacteal feeding to help cleanse the infant's gastrointestinal tract for digestion, quench thirst, flush the bladder, and help the mother to rest after childbirth before BF is initiated.^31^ In Ethiopia, some cultures perceived that complementary food fortifies the child against evil eyes, unlike breast milk.^32^

Similarly, cultural taboos that menstruating women should not carry a child being breastfed exclusively made it difficult for mothers not to wean their infants prematurely. Another taboo related to BF is the belief that a woman who is practicing EBF should not push fire into the cooking stove.^28^ These types of notions made coping with EBF difficult for new mothers who require a lot of support while nursing. Most of these cultural beliefs and practices have remained popular despite modernization and some strategic efforts.

Furthermore, the notions that the mother's diet such as hot food, leafy vegetables, and liquid food may spoil the BM and harm the child if breastfed were rampant in the Gambia. There is also a cultural belief that eating snails while BF will make the infants salivate.^33^ Mothers in Ghana shared the belief that the baby being fussy, not sleeping well and not lasting long between feeds, are evidence that BM is watery and insufficient, hence infants are fed BM mixed with honey and some give a mixture of water and shea butter and herbs to make baby sleep longer.^34^ In Limpopo, it is believed that babies exclusively breastfed may experience colic, leading to excessive crying and fever.^34^ While in Niger‐Delta, grandmothers hold the belief that newborns require sufficient water daily due to the hot weather, and they perceive BM as inadequate for quenching thirst. Consequently, they offer water to the babies after BF to fulfill their hydration needs.^35^

In Tanzania, mothers noted that if the infant burped during the feeding session the mother would have swollen breasts which would cause her pain. It is thus recommended that the mother stop BF until she is treated.^22^ Other common cultural understandings that negatively affected EBF practices include the belief that sick mothers should not BF this is because sick mothers are believed to produce “bad milk” which can transfer illness to the infant through the BM.^5^ Likewise, the notion that mother and child should be separated after birth to allow them to rest is found to be widespread. This has been found to delay the time of BF initiation and promote prelacteal feeding.^36^ The impression that most mothers cannot produce enough BM is equally common in many cultures.^33^

Across the world, and Kenya to be specific, religious beliefs as observed among believers of Islam have largely influenced the length and exclusivity of BF.^23^ Islam prescribes BF and it is described by most Islamic scholars as a highly rewarding act which is a spiritual, religious, and cultural aspect of family formation.^30^ Despite this, the practice of prelacteal feeding was found to be quite common in many Muslim communities.^37^ The belief that colostrum is bad or spoilt BM is equally quite common globally as documented in Nigeria, Kenya, and Egypt.^32^, ^37^, ^38^ Interestingly, some authors reported contradictory beliefs about BF and EBF in the same culture. Notwithstanding the perceived health importance of BF and EBF to mother and child,^20^, ^21^ the early introduction of herbs and solid food in the course of BF is a common child‐rearing practice.^20^ Thus, indicating that other sociocultural factors may be contributing to infant feeding practices in the group.

### 3.3 Sociocultural factors/issues responsible for poor adherence to EBF

This narrative review revealed that on the whole, BF is affected by psychological, cultural, political, and economic factors. These encompass issues like employment, education, place of child delivery, family factors/pressure, as well as cultural values among others.^20^ The determinants of BF practice have been examined through diverse perspectives and methodologies and some researchers revealed that physical or medical reasons are possible denominators for EBF truncation.^39^ Others reported that EBF by mothers living in LMICs is largely influenced by their demography, and social, cultural, and health‐related factors such as personal, intrapersonal, and intrapartum experiences.^32^

#### 3.3.1 Growth of capitalism and commercialization of infant feed

The use of complementary infant feeding before the age of 6 months has been traced to the growth of advertisement campaigns for artificial feeding alternatives which disrupted the adherence to natural feeding crusade.^40^ The invention of food preservation in the 19th century and the approval of formula as a safe alternative to breast milk by American physicians in the 1940s and 1950s are equally major contributory factors. Furthermore, the invention and commercialization of infant formula from the 18th through the 20th century^39^ have also been implicated. Unfortunately, no thanks to globalization, these trends which started in the Western world have spread to the less‐industrialized world through aggressive marketing. This caused a global fall in EBF through the 1970s before a campaign promoting EBF was initiated.^40^ Tracing the origin and causes of poor infant feeding practices, the authors identified that issues like social status, low cost of hiring wet nurses, consciousness about or change in physical appearance, fashion, and disruption of leisure activities (like playing cards and watching theater performances) prevented women from BF their infants in Renascence France. The Industrial Revolution contributed to poor BF, and so did the improvement of feeding bottles and the availability of animal milk.^40^ For instance, in Nigeria, the proliferation of attractive and packaged BM alternatives, as well as marketing strategies adopted by companies producing BM substitutes promoted early supplementation of infant feeding.^41^

#### 3.3.2 Psychosocial factors

A review of previous studies on Nigeria equally indicated that EBF was not practised by mothers because of the discomfort associated with the exercise and the women stopped EBF due to it being very stressful.^42^ Among some women in Uganda and South Africa, a perceived lack of freedom^8^, ^43^ was cited; while change in body appearance discouraged Tanzanian mothers from exclusively BF their infants.^22^ Cognitive factors are further implicated by studies that examined knowledge and perception of EBF. The perception of mothers in Nigeria that the child is always hungry after BF^42^ and the perceived insufficiency of BM for infants made mothers stop EBF.^19^ Knowledge and education on the benefits of EBF promoted the practice among mothers in Ethiopia and Northern Nigeria^19^, ^32^ while inadequate knowledge of EBF influenced its discontinuation in another study in Northern Nigeria and the Democratic Republic of the Congo (DRC).^29^, ^44^ Similarly, low levels of knowledge of BF were negatively associated with EBF in Kinshasha.^45^ On the contrary, knowledge and understanding of the benefits of EBF did not sway its practice among mothers in Ghana.^32^, ^46^ The time of BF initiation was associated with knowledge of the timing of BF initiation in Tanzania.^47^

#### 3.3.3 Biological factors

Biological factors like the sudden onset of new pregnancy made women stop BF in Sokoto.^29^ A similar finding was reported in South Africa.^48^ Natural events like lactation failure, maternal illness,^7^ breast engorgement, sore nipple, and BM insufficiency negatively impacted the practice of EBF hence alternatives like artificial feeding were used to replace natural BF.^49^ Other biological factors are neonatal illnesses (first 28 days).^7^ EBF was found to be difficult when the infant is admitted in a Neonatal Intensive Care Unit (NICU) where replacement feed is usually introduced,^7^ or when the infant has a low birth weight.^50^ Low birth weight, without neonatal illness, did influence EBF negatively in Ghana. Mother's perception of the baby's birth size also influenced the practice of EBF.^50^

#### 3.3.4 Role of significant others

The role of significant others was equally found important in the decision and duration of BF. The attitude of spouses towards BF was identified as a predictor of EBF discontinuation in DRC.^45^ In South Africa, compliance with EBF was enhanced by spousal awareness and perception of the benefits of EBF to the infant.^19^ Similarly, having a BF peer also had positive effects on EBF practice by mothers in South Africa.^51^ In the same vein, belief, knowledge and perception of the major support system influenced BF practices among some Ghanaian mothers.^52^ The availability of family support reportedly encouraged mothers to BF exclusively in a study, especially the influence of grandmothers and husbands.^19^ While poor family support was a major barrier to EBF identified in northern Nigeria.^19^, ^29^ These suggest that people in the social network of lactating mothers are critical to their decision to BF, its exclusivity and duration.

#### 3.3.5 Religious beliefs

The place of social and cultural factors in infant feeding practices are similarly documented by experts who have worked on the subject over time. For instance, a link has been established between religion, the introduction of prelacteal feed, and the decision and duration of BF by researchers in different locations like the Gambia^33^ and Ghana.^52^ In Ghana, a positive association was observed between Islam and Christianity and BF practices.^52^

#### 3.3.6 Cultural norms and traditions

A study in DRC indicated that traditional norms and perceived expectations had strong positive impacts on African mothers' beliefs, attitudes and practices associated with EBF. The authors likewise noted that the beliefs and attitudes held by the social support networks that a woman has had a major influence on her decisions to practice EBF.^53^ Same was true for mothers in Ghana.^54^

Barriers to EBF identified in northern Nigeria are poor family support and cultural practices including traditional uvulectomy on the infant which tends to disrupt the infants' ability to feed properly and some may develop an infection. The notion that the child is old enough to start taking solid food made mothers discontinue BF in north‐western Nigeria.^19^, ^29^ Also, pressures from family and the community, the belief that water aids digestion, family traditions, and the need to adapt the child to other foods invoked a lack of EBF.^32^ The belief that infants breastfed exclusively are susceptible to diarrhea^44^ is another barrier. Traditional beliefs and practices which promote EBF include 40 days of traditional bathing for mothers. During this period, mothers are excused from house chores and other activities.^19^

#### 3.3.7 Sociodemographic factors

Previous studies have shown that the duration and exclusivity of BF are negatively associated with demographic variables like young age, low level of education, being unmarried, low income, out of employment, and parity (first‐time mother). That is, those with these characteristics did not BF their infants for up to 6 months.^45^ However, a study in Kinshasa, DRC, did not find any relationship between the nursing mothers' sociodemographic background, attitude towards BF, time of initiation of BF, and discontinuation of EBF.^45^ Also, in Ghana, the level of education did not translate to the practice of EBF among mothers.^46^ Conversely, the demographic characteristics positively associated with EBF among Ethiopians are occupational status, education, and place of residence. Others include being a housewife, having a personal business, and having a microfinance bank account which were all positive contributors to the practice of EBF in Ethiopia.^55^

In Eastern Nigeria, EBF was higher among multiparous than grand‐multiparous women, it was also higher among mothers who had antenatal registration at the hospital than those registered at the health center.^56^ Sociodemographic determinants of EBF identified in an Ethiopian study are mother's occupation and place of delivery. On the contrary, such variables as gender, marital, education and HIV status, did not influence the practice of EBF.^57^ Another study among working mothers of infants less than 7 months found that their occupational status, nonflexibility of work/nature of work, lack of on‐site childcare support, nonavailability of BF or expressing room, and short maternity leave all discouraged nursing mothers from practicing EBF. Mothers who valued BM highly were determined and resilient and those who were experienced practiced EBF more.^32^

A longitudinal study of determinants of trends in BF practices in Nigeria revealed that EBF was practiced more by educated mothers, mothers from affluent households, frequent users of health services, birthing at a health facility, number of antenatal care visits, and increase in child age reduced the likelihood of EBF.^42^

#### 3.3.8 Health service‐related factors

Among women in Kinshasha, the number of antenatal clinic visits and Baby Friendly Hospital Initiative (BFHI) practices experienced during hospital stays all had a significant influence on the discontinuation of EBF.^45^ In Ethiopia, Biks et al.^55^ reported that receiving antenatal care for the index child and giving birth in a healthcare facility positively influenced EBF.^55^ Lack of information on EBF from healthcare providers and late initiation of BF were also common and found to influence the practice of EBF in Ghana.^46^ In South Africa, the availability of information to aid initiation of BF as well as practical support provided by healthcare providers to address inadequate milk supply aided the practice of EBF and vice versa.^51^ In Ethiopia, counseling about BF before childbirth improved knowledge of EBF whereas counseling did not influence the practice of EBF among the nursing mothers.^57^ While attitude of healthcare providers towards BF and the policies of health facilities are important determinants of EBF in Nigeria.^41^

#### 3.3.9 Early initiation of BF

Early initiation of BF has been shown to promote EBF,^42^ while the long‐time duration for initiation of BF implies that a prelacteal food must have been introduced.^54^ Mother and newborns skin‐to‐skin contact have been found to positively influence the timely initiation of BF in various countries, including Angola, Cameroon, Ethiopia, Guinea, Liberia, Malawi, Mali, Sierra Leone, South Africa, Tanzania, Uganda, Zambia, and Zimbabwe.^58^ Mothers who initiated BF within 1 h of giving birth, and practised skin‐to‐skin contact, tend to practice EBF.^33^, ^58^ However, late initiation of BF was common among those who did not utilize healthcare services and those with lower incomes. Similarly, delivery through cesarean section (CS) reduced the likelihood of initiating BF within 1 h of birth while those who birth at a health facility were more likely to initiate BF within 1 h than those who had home delivery.^42^ Among HIV‐positive mothers in South Africa, fear of HIV transmission, the number of infant feeding counseling sessions attended by mothers, consistency of the information received, number of HCWs involved in care, provision of free infant formula in clinics, and contradictory messages from different HCWs all affected the initiation and compliance with BF.^59^

#### 3.3.10 Individual traits and behavioral factors

Depending on the social context, a multitude of individual traits and behavioral factors determine mothers' feeding decisions.^32^ In Kinshasha, lack of planning on the length of EBF, and a lack of confidence in the ability to BF.^45^

Other factors identified in Nigeria are urbanization, common cultural beliefs about the benefits of colostrum, and sexual practices during BF.^41^ The extended family structure was also found to promote early initiation of BF^20^ and by extension, EBF. Also, the joy of childbirth, personal decisions and other circumstances influenced the duration of BF in Nigeria.^20^

These studies have shown that mothers' compliance with EBF is dependent on sociocultural intricacies which are sometimes out of their control. Biological, economic, and healthcare‐related factors may likewise combine with personal or individual factors to complicate the process.

### 3.4 Feeding‐associated beliefs regarding the need for complementary food

Experts have recommended that complementary food should be fed to babies not later than 26 weeks of life but not before the child is 6 months old.^60^ Others recommended that the age of infant weaning should be between 9 and 12 months or after the child has grown canine teeth.^28^ Notwithstanding the age of weaning, generally, the pattern of infant feeding from age 0 to 24 months is indicated as a major reason for malnutrition. At this age, the nutritional and health benefits of breast milk to infants have been identified. It has been scientifically proven that the likelihood of a child deriving benefits from BF is determined by the time of BF initiation, duration, and age when the infant is weaned.^38^ However, the decision and time of child weaning are shaped by culture and traditions among other social variables like cultural beliefs.

#### 3.4.1 Cultural beliefs about weaning and complementary feeding

Studies on beliefs about infant complementary feeding in Uganda found many taboos and cultural perceptions which have a significant influence on weaning and infant feeding practices. Shifting from breast milk to other diets has been linked to the notion that breast milk is an incomplete food that does not lead to weight gain.^28^ A similar belief has been reported among some Nigerian mothers who perceived that formula makes babies grow bigger unlike breast milk.^61^ The Ugandan study also found that mothers held the notion that EBF makes babies susceptible to illnesses, hence babies are weaned before they are 6 months old. More like this is the notion held by mothers‐in‐law and significant others about child weaning which were found to have equally influenced the time and type of complementary food given to children.^28^

Mothers in DRC believe their infants are old enough to start complementary food (porridge) as it is perceived that they do not get enough milk to make them full at a certain age. They believe that BM is hot so they introduce water to aid the digestion of human milk. The notion that water is life, and a child must be given water was widespread in the group. Water is also used as an antidote to hiccoughs.^44^ Some mothers have the belief that complementary feeding is needed for a quick and easy adaptation of babies to alternative meals despite their understanding that EBF is good for both mother and child.^20^ Common social norms, taboos, cultural beliefs and expectations about BF, infant feeding and weaning in society reportedly led to stigma, pressure and negative comments thereby forcing mothers to wean their babies earlier than planned in Uganda.^28^

### 3.5 Policies/programs and best practices that work to promote EBF

Based on the available evidence to support the health benefits of BF, a number of local, national, and international campaigns have been initiated since the 19th century to promote BF, both its duration and exclusivity. In 1991, the WHO introduced the BFHI to protect, promote, and support BF across the world.^62^ The initiative was presented at the summit of experts from the WHO and the UNICEF. The INNOCENTI Declaration was proposed at the Policy Makers Meeting on BF. The declaration was made to protect, promote, and support BF. It prompted BF policies around the globe^41^ and was adopted by many governments.^45^ According to Yotebieng,^44^ the BFHI was built on the “Ten Steps to Successful Breastfeeding” which was introduced in 1990 to promote EBF around the world. According to the document, only health centers that are compliant with supportive steps designated as “baby‐friendly.”

The BHFI document indicated that BF should be initiated early, babies should be breastfed on demand, and no prelacteal feeding should be introduced to infants for 6 months without medical recommendation.^31^ After 6 months of EBF, BF may be complemented with semisolids and solid meals for up to 2 years.^29^ Another policy that has facilitated BF is the Convention on the Rights of the Child (CRC) to which many governments were likewise signatories. Subsequently in 2004, WHO and UNICEF both unveiled the Global Strategy for Infant and Child Feeding. After signing the declaration, Nigeria and 11 other countries were selected to start the BFHI.^41^ Most African countries revealed their support for the international declaration and convention in December 1990 when the government promulgated a decree to ban the importation, marketing, distribution, and sale of human milk alternatives without the approval of designated authorities. Many countries also introduced the National Breast Feeding Policy which is to protect, support, and promote EBF.^41^ Unfortunately, the prevalence of EBF in Africa has not been as encouraging as expected.

The South African government introduced the Tshwane Declaration of Support for Breastfeeding as an instrument to change infant feeding, promote EBF and eliminate complementary feeding by HIV‐positive mothers in the country.^48^ The initiatives introduced in DRC to promote EBF are the national code of marketing alternatives to BM as well as the Baby Friendly Communities initiatives. Although, as of 2010, the rate of BF initiation was remarkable, however, only a small percentage of women EBF their infants.^45^

In Ghana, the policies to promote BF include infant and young child feeding (IYCF) and BFHIs. Peer counselors were utilized in communities where child delivery mostly takes place at home. Another program introduced in developing economies is the Integrated Management of Childhood Illness. Others are Mother‐to‐Mother (M2M) peer support initiative first introduced in South Africa. It is also known as “Mentor Mother.” It involves engaging the services of mothers who are living with HIV to counsel, advise, and support nursing mothers and pregnant women living with HIV.^63^ Another policy in Africa is the counseling sessions organized for lactating mothers as reported in Ghana. This entails training and educating mothers on BF.^46^

Under the authority of the Ethiopia National Nutrition Program, the Lifecycle Approach was introduced to promote BF of infants within the first hour of birth, EBF for 6 months and satisfactory complementary feeding.^57^ Although, some of these interventions did not yield the desired outcome many have, however, helped increase the rate of BF and EBF in the population.

## 4 CONCLUSION

EBF is highly recommended for newborns because of its many benefits. Unfortunately, the practice of EBF is relatively low in several countries. There are many context‐specific challenges militating against EBF in Africa. The adoption or nonadherence to EBF is a sociocultural issue in Africa. Cultural norms, beliefs, and traditions related to EBF exist in every culture. This explains why EBF is strongly rooted in culture and is a culturally determined behavior. There are misconceptions regarding colostrum as dirty and harmful, hence, prelacteal feeding is initiated. The availability of family support (especially from grandmothers and husbands) reportedly encouraged EBF. The duration and exclusivity of BF are negatively associated with demographic variables like young age, low level of education, being unmarried, low income, out of employment, and parity (first‐time mother). While there have been some efforts and policies to improve EBF, it is important to consider context‐specific challenges and sociocultural factors. There is a need for more deliberate efforts to encourage mothers through the implementation of effective best practices concerning EBF in Africa.

## AUTHOR CONTRIBUTIONS

**Jimoh Amzat**: Conceptualization; investigation; funding acquisition; writing—original draft; methodology; validation; visualization; writing—review and editing; software; formal analysis; project administration; data curation; supervision; resources. **Kafayat Aminu**: Investigation; writing—original draft; methodology; validation; visualization; software; formal analysis; data curation; resources. **Brisca Matankari**: Writing—original draft; resources; formal analysis; data curation; investigation. **Abbas Ismail**: Resources; writing—review and editing. **Bello Almu**: Resources; writing—review and editing. **Kehinde Kazeem Kanmodi**: Resources; writing—review and editing.

## CONFLICT OF INTEREST STATEMENT

Kehinde Kazeem Kanmodi is an Editorial Board member of Health Science Reports and a coauthor of this article. To minimize bias, they were excluded from all editorial decision‐making related to the acceptance of this article for publication. Other authors have no conflict of interest to declare.

## TRANSPARENCY STATEMENT

The lead author Kehinde Kazeem Kanmodi affirms that this manuscript is an honest, accurate, and transparent account of the study being reported; that no important aspects of the study have been omitted; and that any discrepancies from the study as planned (and, if relevant, registered) have been explained.

## Data Availability

Data sharing is not applicable to this article as no new data were created or analyzed in this study.
